# The influence of climatic and environmental variables on sunflower planting season suitability in Tanzania

**DOI:** 10.1038/s41598-023-49581-5

**Published:** 2024-02-16

**Authors:** John Beteri, James Godfrey Lyimo, John Victor Msinde

**Affiliations:** 1 Institute of Development Studies (IDS), University of Dar es Salaam, Dar es Salaam, Tanzania; 2 Institute of Resources Assessment (IRA), University of Dar es Salaam, Dar es Salaam, Tanzania

**Keywords:** Climate-change mitigation, Agroecology, Ecology, Climate-change ecology

## Abstract

Crop survival and growth requires identification of correlations between appropriate suitable planting season and relevant climatic and environmental characteristics. Climatic and environmental conditions may cause water and heat stress at critical stages of crop development and thus affecting planting suitability. Consequently, this may affect crop yield and productivity. This study assesses the influence of climate and environmental variables on rain-fed sunflower planting season suitability in Tanzania. Data on rainfall, temperature, slope, elevation, soil and land use/or cover were accessed from publicly available sources using Google Earth Engine. This is a cloud-based geospatial computing platform for remote sensed datasets. Tanzania sunflower production calendar of 2022 was adopted to mark the start and end limits of planting across the country. The default climate and environmental parameters from FAO database were used. In addition, Pearson correlation was used to evaluate the relationship between rainfall, temperature over Normalized Difference Vegetation Index (NDVI) from 2000 to 2020 at five-year interval for January-April and June–September, for high and poor suitability season. The results showed that planting suitability of sunflower in Tanzania is driven more by rainfall than temperature. It was revealed that intra-annual planting suitability increases gradually from short to long- rain season and diminishes towards dry season of the year. January-April planting season window showing highest suitability (41.65%), whereas June–September indicating lowest suitability (0.05%). Though, not statistically significant, rainfall and NDVI were positively correlated with r = 0.65 and 0.75 whereas negative correlation existed between temperature and NDVI with r = -− 0.6 and − 0.77. We recommend sunflower subsector interventions that consider appropriate intra-regional and seasonal diversity as an important adaptive mechanism to ensure high sunflower yields.

## Introduction

Apart from environmental factors, more specifically soil characteristics, suitability of crop planting seasonality in rain fed crop production scenarios is largely determined by climatic conditions in many parts of the world^1–3^. While rain is responsible for water availability to the crops, temperature controls the growing length, phonological development and the productivity of crops^4^ with radiation ensuring energy supply. If these important climatic aspects are not optimally met, the crops may fail to germinate and establish properly, and hence poor production rate. The ongoing climate change, have severely impacted rain fed crop production dependent areas across different parts of the world^5–7^. The main climatic threats that alter crop location specific planting season are those related to decrease in rainfall amount and frequency, and temperature rise. Ultimately, these changes lead to reduction of yields for several annual crops^1,6,8^. For instance, shifting of precipitation and temperature patterns will cause an increase of unpredictability of growing dates and increase the direct heat and water stress to some grain crop varieties in western and southern hemisphere^9–11^. Nevertheless, attenuating these severe impacts of climate change, farmers need to select appropriate suitable sowing/or planting dates, breeding drought tolerant varieties and shifting growing areas so as to ensure high productivity of crops.

In East Africa, climate change impacts have created unpredictability environment in terms of what crop, when and where to be planted. In case of sunflower, farmers in this region are confronted with a risk of dry spells and puts their crops under sensitive heat and water stresses^12–14^. Farm preparation commence prior to onset of seasonal rains whereas planting season window is mainly scheduled following the rain onsets. As a result, sunflower crop face water and heat stress at their critical development stages such as flowering and grain filling^12^. Moreover, delayed rain onsets in the region leads to shortened growing season window consequently leading to massive crop failures^12,15,16^. Also, environmental alterations due to climate change cause yields reduction due to increase in crop diseases, pests and emergence of new weeds.

In Tanzania, temperature rise, shifts and unpredictable rainfall patterns expose farmers to uncertainty of planting dates and crop managements^14,17^. Planting seasonality studies indicated an increase in intra-seasonal and annual temperature trends and variability in several sunflower currently cultivated areas. Future climate projections indicate that temperature will rise by 2.8 °C and 2.5 °C in the sunflower growing areas of western and eastern parts of Tanzania respectively by 2050 due to climate change and variability^14,18–20^. As rain intensity is expected to decline with change in its distribution patterns across many regions of the country, inappropriate methods of timing planting seasons can subject farmers to low production yield. Furthermore, climate change is expected to affect crop growth duration over different environmental characteristics hence affect yields^21,22^. Assessment of suitability of planting season window based on different climatic and environmental characteristics is imperative to help farmers in Tanzania sow their crops during appropriate planting season windows to utilize the varied climatic seasons across the country to rise sunflower productivity.

Currently, smallholder farmers who are the main sunflower producer in many parts of Tanzania use traditional knowledge and experiences in timing their planting dates^17,23–25^. One of the problem associated with these mechanisms is that they are neither systematically documented nor scientifically investigated. In addition, they can give unreliable information and finally mislead farmers in planning appropriate planting seasons. In some areas dry soil planting has been commonly used albeit with challenges from insects that tend to eat the sown seeds before germination^25,26^ and washing away of seeds by heavy rains during onset times^27^. Moreover, climate change continues to make the planning mechanisms of suitable planting dates more unrealistic due to shift in rainfall patterns with increased unpredictability and temperature rise effects. It has so far not been well established how farmers determine the suitable planting season for sunflower production considering variations in climate and associated environmental characteristics in Tanzania. To avoid these challenges posed by traditional mechanisms, farmers need to make use of consistent mechanisms that enable fully and reliable identification of the seasonal distribution of planting season in the country.

The objective of this study is thus to investigate the influence of climatic and environmental variables on suitability of sunflower planting season in Tanzania by using Google Earth Engine (GEE). GEE provides open access, flexible and robust crop climatic and environmental datasets that enables derivation of dynamic maps even at regional or national level. The study hypothesized that suitability of planting season is mainly the function of climate and environmental factors. The hypothesis was tested by using Pearson correlation method. Tanzania Sunflower Production Calendar of 2022 was adopted to mark the actual start and end limits of growing season of sunflower under rain fed scenario.

## Materials and methods

### Description of the study area

**Figure 1:**
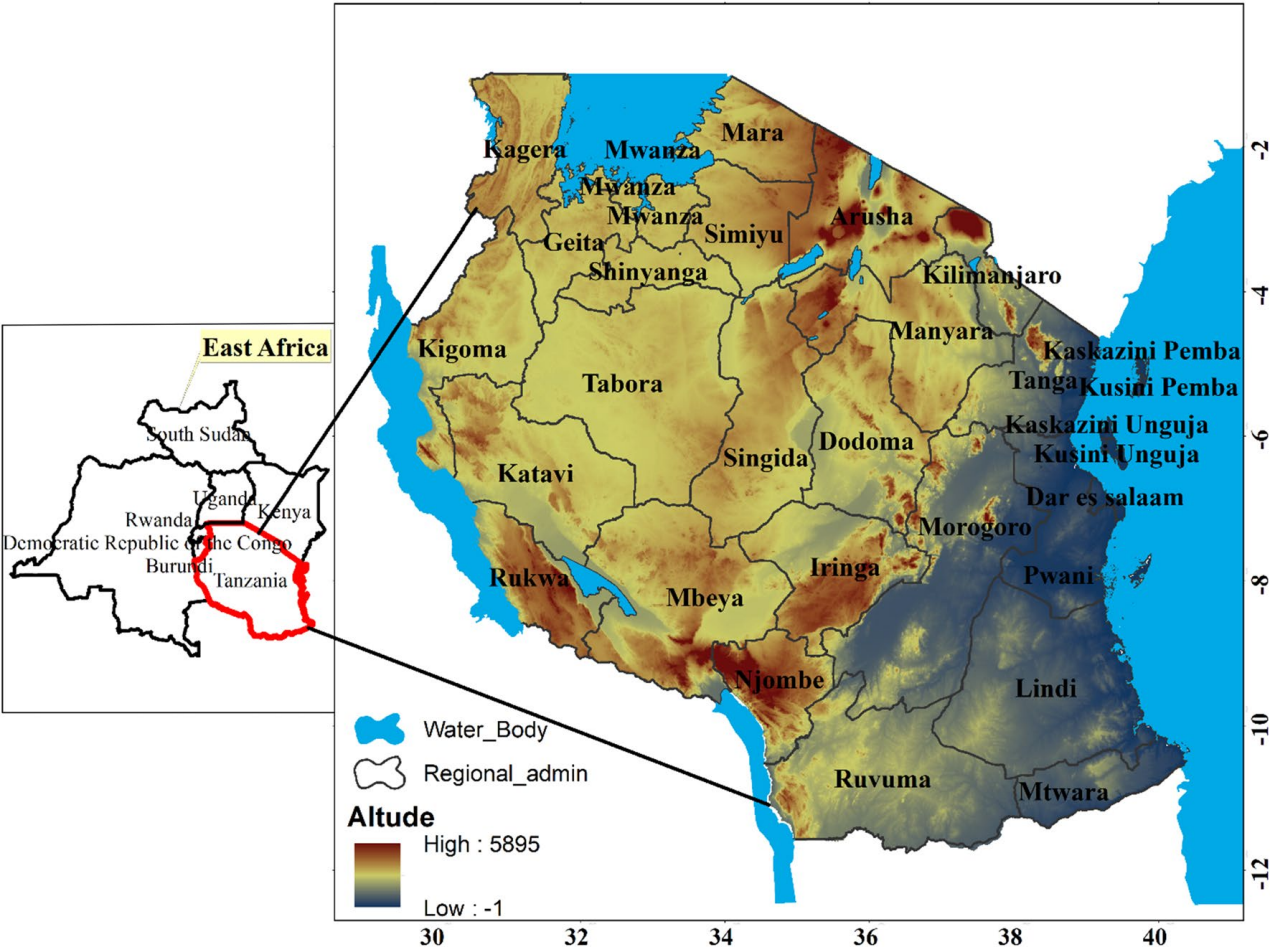
Map of the study area.

Tanzania is located in East Africa, between 29–41°E and 1–12°S (see Fig. 1). It borders Indian Ocean in the East, Malawi and Zambia in South-West, Rwanda, Burundi and Democratic Republic of Congo in West, Kenya and Uganda in North and Mozambique in the South^28^. The country is endowed with diverse topography, climate and soil types. According to the recent 2022 census the country has a population of about 61.5 million people^29^. The country has a total area of 945,087 km^2^ with 883,749 km^2^ and being the land area whereas 59,050 km^2^ is covered by inland water and the Indian Ocean^20^. Agriculture is the main stay of the country’s economy of which 6% of the land is under sunflower cultivation, with the central zone producing 61% of all sunflower produced^14^. Tanzania has variation of soil types across geographical locations. Sandy soils are merely predominant in the coastal regions; red soils are commonly found in the central plateau regions with granite soils dominating in the near Lake Victoria regions. Ironstone and volcanic soils are found in western and northern highland regions.

Climatically, the country has variable climatic characteristics; for instance coastal regions have an average temperature of 27 to 29 °C whereas the central, western and northern regions have temperature ranging between 20 and 30 °C based on the World Bank Climate Change Knowledge Portal (2021), (https://climateknowledgeportal.worldbank.org/country/tanzania/climate-data-historical). Due to complex nature of topography and landforms, Tanzania experiences unimodal and bimodal rainfalls^30^. The unimodal means only one rainfall peak per wet season without an alteration of humid and dry month, whereas bimodal is characterised by two wet seasons separated by a dry season. Bimodal rainfalls occur in the northern-eastern highlands, coastal areas and Zanzibar Isles with rainfall ranging from 750 to 1200 mm per annum. March, April and May (MAM) is considered as a long rains while September, October and November (SOND) is a short rains^31^. The central, southern, western, and south-western highlands receive unimodal rainfalls ranging from 300 to 2000 mm^31^. The Inter-Tropical Convergence Zone (ITCZ) is the main driver of the rainfall distribution pattern in Tanzania^16–33^. Sunflower is grown in almost all the ecological zones of the country, with concentration being in the central zone.

### Input datasets

Planting season window, in this study selected environmental and climate datasets which were accessed from different online sources using GEE, a cloud computing application at various sources (see Table 1). The detailed explanation of variables is provided below.

**Table 1:** List of environmental and climatic variables that were extracted from GEE.

Dataset	Source	Product	Range	Temporal	Resolution
Rainfall	CHIRPS	Chirps monthly	2000- current	3-day	5.5 km
Temperature	NASA-MODIS	MOD11A2 v006	2000-current	16-day	1 km
NDVI	NASA-MODIS	MOD13Q1 v006	2000-current	16-day	250 m
Agro-LULC	NASA-MODIS	MCD12Q1 v051	200–2013	Annual	500 m
		MCD12Q1 v006	200–2018	Annual	500 m
	ESA	Glob Cover2009	2009	Annual	500 m
	USGS	GFSAD1000	2000	Annual	1 km
Soil properties	OpenGeoHub	OpenLandMaps	–	–	250 m
Elevation	NASA/NGA/DLR/DET/ASI	SRTM	2000	–	30 m

### Slope data

The slope may prohibit water infiltration into the soil thus affecting crop growth. Sunflower prefers areas with an altitude of 2600 m mean sea level in tropics. It can grow well below 1500 m with slope ranging from 8.3% (FAO, 2000). In this study, slope and elevation data were accessed by using google earth engine from USG Shuttle Radar Topography Mission (SRTM) released in 2000 at 30 m ground resolution^34^.

### Land use/cover data

Sunflower grows well in steppe, humid and semi-arid areas characterized by natural woodland, thickets, grassland, shrubs, and abundant forests. Hence, land use/cover is a key input in the study. Data for this variable was extracted from Globe Cover ESA/GLOBECOVER at 300 m, Moderate Imaging Spectrometer (MODIS) Land Cover Type 500 m, V051 (ii) and V006 (iii) and Global Food-Support Analysis Data for cropland extent at 1 km^35^. With the use of GEE application waterbodies within the study area were masked out, hence not considered in the analysis since these were considered as irrelevant and unsuitable areas.

### Climate data

Change in temperature and rainfall patterns can affect crop growing season and its pattern too. The variability of rainfall and average temperature can lead to changes in growing season duration and increased crop and prematurity^36–39^. Low rainfall results to poor crop development^38,40^. Sunflower flourishes in low and moderate humid, tropical wet and dry, semi-arid or steppe, subtropical humid, subtropical dry summer, subtropical dry winter, temperate with dry winters and temperate climate with optimal temperature between 17 °C and 34 °C. It requires rainfall amount around 300 and 1600 mm (FAO, 2000). Daily (minimum & maximum) rainfall data were accessed from the Climate Hazard Group Infrared Precipitation with Station, (CHIRPS) at 5.5 km resolution^41^. Similarly, Google Earth Engine was also used to obtain data on minimum and maximum temperature (°C) at 8 days temporal resolution from MODIS Terra Land Surface Temperature product^42^.

### Soil data

Sunflower thrives in sand, silt and clay loam soils, with pH of 6 to 7.5. In addition, the crop grows well in areas with well-drained soil with dry spells, medium and light textures, low salinity of < 4 dS/m, bulk density of 1.4 g/cm^3^, 16% soil water content and 13.3C/kg organic matter and 50–150 cm soil depth (FAO, 2000). The soil characteristics of relevance to this study included soil pH, organic carbon (c/kg), bulk density (g/cm^3^), soil water content, taxonomy groups (kPa) and soil texture (sand/silt/clay). Data on these parameters was obtained from the OpenGeoHub LandGIS^43,44^. This uses an automated technique by enabling farmers and researchers to collect and measure soil properties from field samples for regular improvement and is often updated in GEE. It is available at 250 m spatial resolution (Table 1).

### Normalized different vegetation index data

Normalized Difference Vegetation Index (NDVI) was initially developed in 1973 by a research team at Texas A&M University. It is the spatial technique used in crop managements to evaluate biomass content of plants and /or crops on time-series manner. In this study, vegetation indices data was obtained from NASA MODIS^45^ and used to quantify crop production in the study area based on planting suitability windows.

### Tanzania sunflower production calendar

Sunflower is the annual crop whose growth cycle ranges between 90 and 160 days (FAO, 2000). Therefore, cropping calendar is important as it allows farmers to plan from land preparation to cropping and harvesting stages. Tanzania sunflower production calendar of 2022^46^ was adopted to set the start and end limits of the planting dates over various inter regional and/ or regional climatic conditions.

### Methods

The Google Earth Engine (GEE) was introduced by Google in 2010 at the International Climate Change Conference in Cancún, Mexico^47^. GEE is an open access web-based platform that allows access, computation and/or processing of large scale-satellite images from many computer-servers in Google’s data centers^48^. In this study, the suitability of sunflower planting season windows in Tanzania was carried out under six procedures using scripts modified from Ref.^49^. First, Tanzania was set into the application as the study area boundary. Second, the platform was set to acquire publicly accessible and high spatio-temporal resolution global climatic and environmental datasets; such as UCSB Climate Hazards Group precipitation^41^, NASA MODIS land-cover type (MCD12Q1)^35,50^, NASA MODIS temperature (MOD11A2)^42^, NASA/USGS cropland extent (GFSAD)^35^, ESA land-cover type (GlobCover)^51^, NASA MODIS LST(MOD13Q1)^45^, OpenGeoHub/LandGIS soil properties^43^, it include soil properties such as organic carbon content, bulk density, pH, soil water content, sand/silt/clay fraction and biome/taxonomy groupings and NASA/NGA/DLR/DET/ASI elevation (SRTM)^34^ as explained in details in the input data section and shown in Table 1.

Third, masking the area under agriculture and cropland; MODIS/006 (MCD12Q1)^35,50^ and UMD/hansen /global forest (2015) were used as input datasets for creating layers for masking out waterbodies in the study area since the study considered them as permanent non suitable areas. Meanwhile, NASA/USGS/GFSAD1000_V1^35^ and ESA/GlobCover^51^ were used to delineate agriculture and cropland extent.

Fourth, 2000–2001 and 2001–2002 were defined as the temporal aggregation ranges (years) followed by crop growth season duration in month/days (MM-dd) which was established to meet the seasonal duration of sunflower crop under rain fed scenario. In Tanzania, rain takes place during September, October and November (SON) as short rains, and from March, April to May (MAM) as long rains, respectively. This shows wrapping/or an overlap of sunflower growing seasons over the next year. Therefore, if data are aggregated based on single year it is possible that the results could be product of two distinctive growth seasons. To address this, we specifically adopted the Tanzania sunflower production calendar of 2022^46^ to customize the start and end of sunflower planting season in Tanzania based on geographical patterns of climatic and environmental characteristics. Planting season were made to wrap over the next year, in order to connect from one year to the following year. Therefore, a set of 4-month moving planting season windows for 12 months from two different sequential time ranges were used namely; from 2000 to 2001 for October-January, November-February, and December-March, whereas 2001–2002 was used for January-April, February-May, March-June, April-July, May–August, June–September, July–October, August-November, September-December.

Fifth, defining sunflower crop requirements or thresholds; each crop requires specific climatic and environmental characteristics to thrive. In this study, FAO ECOCROP database (1991), available from https://gaez.fao.org/pages/ecocrop-find-plant was used to customize sunflower growth season requirements in terms of rainfall, temperature and other environmental ranges. The ecological crop parameters in the ECOCROP be used to customize thresholds of possible suitable crop growth season of any crop^52^ with albeit differences observed among crop species^53^.

Lastly, after all the input datasets met ten raster layers in three types were loaded in the GEE application interface. The first layers displayed include mean seasonal temperature and rainfall, mean seasonal NDVI. The second layers consisted of temperature, rainfall, and combined suitability boolean map layers. The third map layer include the combined crop suitability of planting season characterized into suitable or not suitable classes based on temperature and rainfall input. The final combined crop suitability displays two values, 1 and 0. 1 represents area with suitable planting season whereas the value of 0 represents non-suitable planting season. The maps and figures were plotted in R studio software.

### Temporal planting season suitability ranking

The study also evaluated temporal comparison between sunflower suitability and non-suitability windows in the entire year. This was important so as to understand the ranks and direction of sunflower production season in the country. Thus, percentage of each planting season window was computed by taking the total number of suitable pixels in square meters over total area of the country multiplied by 1,000,000. The rationale was to convert square meters into square kilometers.

 Hence, the following equation.

1 $$e= \frac{\left(a*b\right)}{d}*1000000$$

where, $$a=\mathrm{size \,of\, one\, pixel\, in\, square \,meter}$$, $$b=\mathrm{total \,number \,of \,pixels\, of \,suitable}/\mathrm{non\, suitable\, planting\, season \,in \,square \,meter},$$ $$c=\mathrm{total\, size \,of \,suitable\, land\, in\, square\, meter}$$, $$\mathrm{thus\, }c=a*b$$, $$d=\mathrm{total \,size \,of \,Tanzania\, land\, in\, square \,meter}$$, $$e=\mathrm{fraction\, of}$$ $$\mathrm{suitable\, land\, in \,square\, meter}$$.

After obtaining the total size of suitable and non-suitable land for each planting season at countrywide level, the next step was to indicate the simultaneously highest planting suitability and non-suitability in a year in order to highlight their ranks. In this case, both variables were converted to the same scale. The values for suitable and non-suitable land were normalized between 0 and 1 value using the equation: $zi=\left(\text{Xi-Min(x}\right))/(Max\left(x\right)-Min\left(x\right).$where $zi=the \,ith \,normalized\, value\, in\, the\, dataset$, $xi=\text{the\, ith \,value\, in \,the\, dataset}$,$\left(\text{Min(x}\right)=the \,minimum\, value\, in \,the\, dataset$, $Max=the\, maximum \,value \,in \,the \,dataset$. Then after, the ranks were presented through mirrored histograms and radial graphs R studio in ggplot2 package^58^.

### Pearson correlation method

The relationship between the average values of NDVI and climate factors from 2000 to 2020, was evaluated by using Pearson correlation approach. The interval of five years over two planting windows was adopted. The selected planting suitability windows were; January-April, representing good planting window and June–September represented low planting suitability, respectively. The NDVI, rainfall and temperature values for each variable were retrieved by GEE and converted into excel data sheet. These variables were selected because they can affect positively and/ or negatively crop sowing dates, planting acreages and ultimately lower the crop yields^37,38,59^. Pearson’s correlation method in R studio was carried out so as to know their correlational strengths. This statistical analysis is recommended in analyzing statistical variables (data) which have time intervals with suspected linear relationship between variables and has been adopted elsewhere e.g. Ref.^60^. The *r* values were used to judge the strength of relationship with *r* given a value of 1 or − 1. The value 1 indicates strong positive association between the two variables and -1 indicates strong negative association whereas 0 denotes no relationship between the variables. The strengths of correlation were categorized based on Ref.^61^ that 0.00–0.19 (very weak), 0.20–0.39 (weak); 0.40–0.59 (moderate); 0.60–0.79 (strong); and 0.80–1.0 (very strong). The following equation was used to compute this association.

2 $$r=\frac{n(\sum xy)-(\sum x) (\sum y)}{\sqrt{[n\sum {x}^{2}-(\sum {x)}^{2}}][n\sum {y}^{2}-(\sum {y}^{2})}$$

where, ${\text{r}}=$ Pearson coefficient; ${\text{n}}=$ number of the pairs of the variables; $\sum {\text{xy}}=$ sum of the products of the paired variables; $\mathrm{\Sigma x}=$ sum of the x scores; $\sum {\text{y}}=$ sum of the y scores; $\sum {{\text{x}}}^{2}=$ sum of the squared x scores; and $\sum {{\text{y}}}^{2}=$ sum of the squared y scores.

## Results and discussion

### GEE assessment results

**Figure 2:**
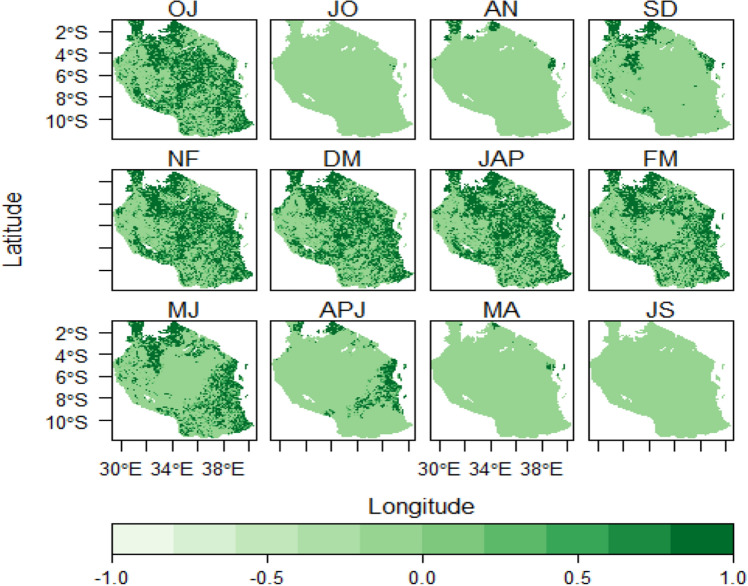
Sunflower planting seasonality patterns in Tanzania on a multiple temporal dates; Simulated time ranges; 2000–20001 and 2001–2002. Note; the white patches denote water bodies.

The results from GEE analysis yielded a twelve 4-months moving windows of sunflower planting season in Tanzania (see Fig. 2). Results shows that suitability of planting season windows are influenced more by rainy season distribution patterns in Tanzania. The spatial pattern of planting season windows in October-January, November-February and December-March corresponded to short rainy seasons that span from October, November to December (OND) with average suitable area of 39.58%, 40.41%, 40.97% and 39.58%, respectively. Most suitable areas in this period are mainly distributed in the central corridor, southern highlands, coastal regions, areas around Lake Victoria and some parts of Zanzibar Islands. Earlier planting date studies in selected sub-Sahara African regions (Tanzania, Malawi, Uganda, Ethiopia, Zambia and Zimbabwe), pointed out that planting season is determined by rainfall distribution within the season and year-to-year variability in rainy onsets would imply shifting of planting dates^62–66^.

On the other hand, during the long rains season planting suitability tends to expand more geographically with January-April recording the highest spatial coverage score (42.04%) out of all planting season windows produced. The highest score of the planting window in January-April is due to the fact that during this particular time long rains tend to be more established thus ensuring broader production area and higher yields. Stable and optimal rainfall can ensure wider suitable production area and higher sunflower yield in the study area followed by February-May, March-June and April-July with 35.26%, 26.28% and 9.25% (see Fig. 2). However, least planting suitability was revealed from May–August, June–September and July–October with 0.72%, 0.05% and 0.001%, respectively (see Figs. 2 and 6). Therefore, the suitability of sunflower planting windows decreases as one moves towards the dry season of the year when water shortage becomes critical and cannot allow seeds germination especially in the main sunflower growing areas. Based on these findings one can learn that generally is usually safe for sunflower farmers to plant crops after the rain has established to ensure maximum yields^17,24^.

**Figure 3:**
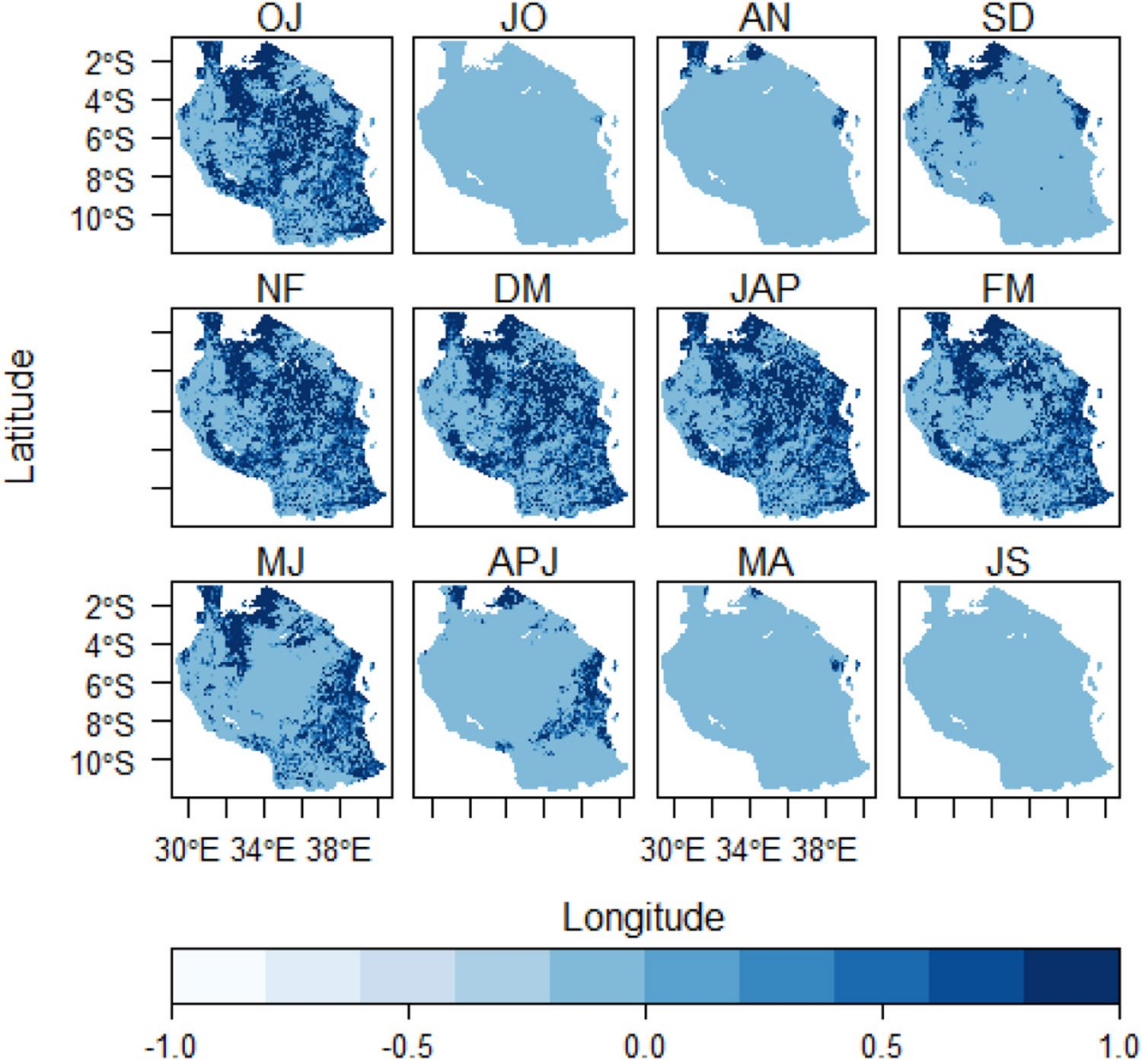
Rainfall distribution patterns over the 4-month planting seasons in Tanzania. *Note;* the white patches denote water bodies.

The new season of planting based on these results connect to next year from August-November and September-December (Figs. 2 and 3). Results in this study corroborate with previous related studies where variations of rainfall seasonality and temperatures have been shown to affect spatial pattern of the suitability of growing seasons for many annual crops and ultimately impacting their yields^15,65,67^. From agronomical perspective this particular finding may help policy makers and agricultural extension workers in Tanzania to determine crop and locational based appropriate planting dates that will enable farmers to grow their crops and maximize yields. It should however be noted that other households’ and farm-specific variables related to, socio-economic and agronomic factors such as, seed availability, planting technology, farm management practices can as well affect timing of planting season suitability windows.

### Rainfall suitability distribution patterns

Rainfall distribution patterns were analyzed for each season window. The results indicate that rainfall suitability patterns corresponded spatially to planting season windows both during the short and long term rain seasons (Figs. 2 and 3). Higher rainfall suitability in October-January, November-February and December-March indicated in (Fig. 3) spatially correspond with planting suitability which is mainly distributed in areas around Lake Victoria, central, southern highlands, coastal regions and Zanzibar Isles. The planting suitability distribution is more related to the start of short rain seasons in these areas. It was indicated that January-April and February-May had highest rainfall suitability occupying areas around Lake Victoria, central, east-southern highlands regions, central, coastal areas, parts of Zanzibar Isles. From February-May to May–August season windows rain tends to decline (see Figs. 2 and 3). The least rainfall suitability begin from May–August, June–September, July–October to August-November. These results may help farmers to critically identify seasons that are quite vulnerable to dry spells and therefore be in position to adopt sustainable adaptive strategies in their specific regions. Closely related studies conducted elsewhere in Africa that associated geographical planting season with rainfalls reported similar findings^3,67,68^.

### Temperature suitability distribution patterns

**Figure 4:**
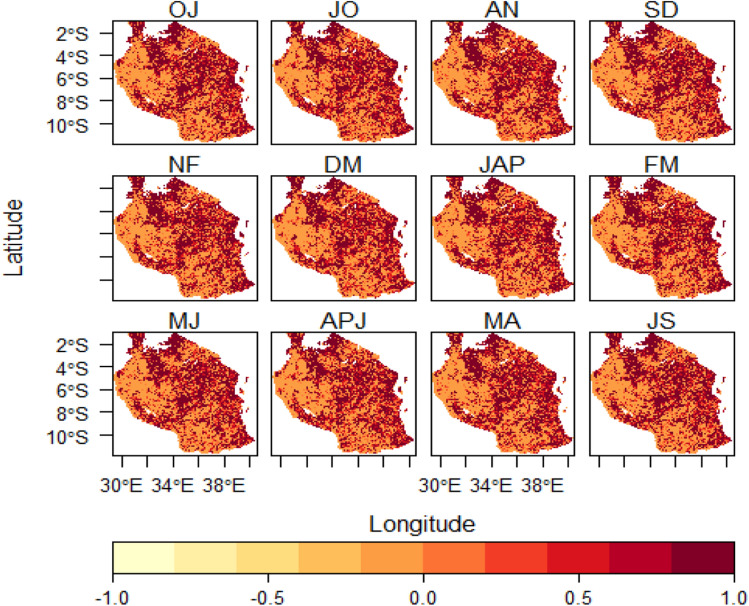
Temperature distribution patterns over the 4-month-planting season window in Tanzania. Note; the white patches denote water bodies.

It was found that, temperature suitability tends to meet sunflower planting requirements or preferences for all windows in many areas in Tanzania especially in the central, areas around Lake Victoria, east-southern highlands, coastal regions and the Zanzibar Isles (Fig. 4). This doesn’t overrule the reality that these places have variations in their own daily temperature ranges depending on the prevalent seasonal weather and climatic condition.

Generally, temperature suitability pattern for each planting window seemed to have high geographical coverage regardless of the prevailing rainy season present. This was different as compared to rainfall suitability whose spatial patterns only depended on rainy season of the respective area. The same observations were made in the study by^69^ in Northern China where temperature was found to meet the potato panting and growth requirements almost for all seasons for five decades. This is in line with the studies conducted on global scale by^1,3^ that planting suitability for many crops especially under rain-fed scenarios is estimated from climatic condition in different part of the world, so Tanzania cannot is inclusive.

### Planting season suitability and non-suitability order

**Figure 5:**
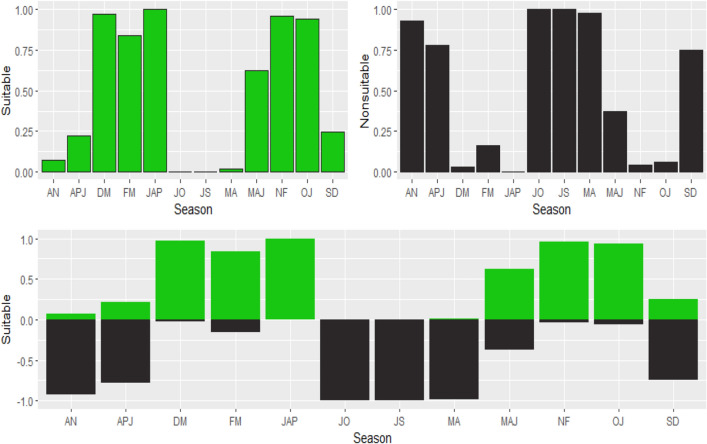
Mirrored bar plot showing seasonal variations in suitable *(green)* and non-suitable planting season *(black)*. *AN* August-November, *APJ* April-July, *DM* December-March, *FM* February–May, *JAP* January-April, *JO* July–October, *JS* June–September, *MA* May–August, *MAJ* March-June, *NF* November-February, *OJ* October-January and *SD* September-December.

**Figure 6:**
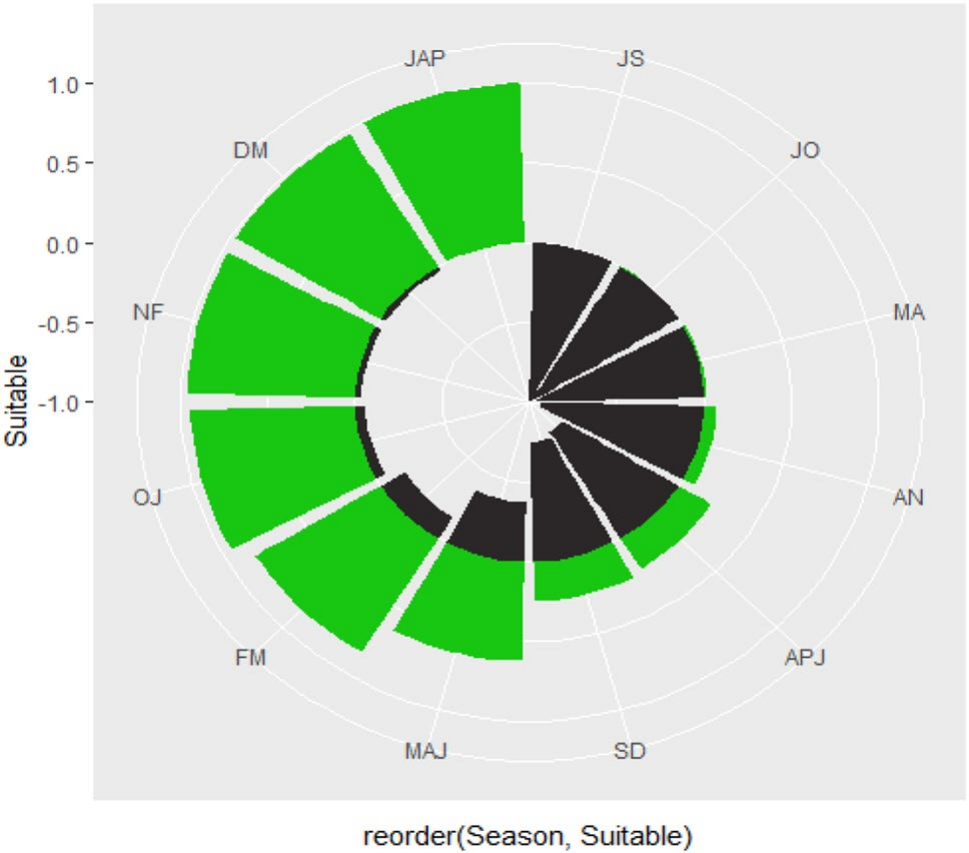
Radial bar plot showing a systematic suitable planting window of sunflower (green).

The study also evaluated the temporal gradual change of planting suitability depending on the rainy seasonality across all 12 planting windows simulated. The mirrored bar shown in (Fig. 5), depicts the inverse or comparison between suitable and non-suitable planting window at the same season with each variable having different units. The radial bar in (Fig. 6), highlights that January-April, December-March, November-February, October-January, February-May and March-June, respectively are markedly with highest planting suitable season windows in Tanzania. This coincides with stable rainfall in many parts of the country including central, areas around Lake Victoria, east-southern highlands, coastal regions and the Zanzibar Isles (Fig. 3). Meanwhile, September-December, April-July and June–September represent low production season in a year. Non-suitable planting windows shown in (Fig. 6) match with dry condition occupying almost the entire country during at this period (Fig. 3). Generally, farmers in Tanzania may detect an appropriate planting season based on their local climatic and environmental backgrounds under rain fed production (Figs. 3, 5 and 6). The practical relevance of this finding is that, farmers may have high degree of precision to ensure survival of their crops since recommended crops’ reproductive/growth phases will correspond with existing suitable climate and environmental factors.

### Statistical relationship between climate factors and sunflower NDVI

**Figure 7:**
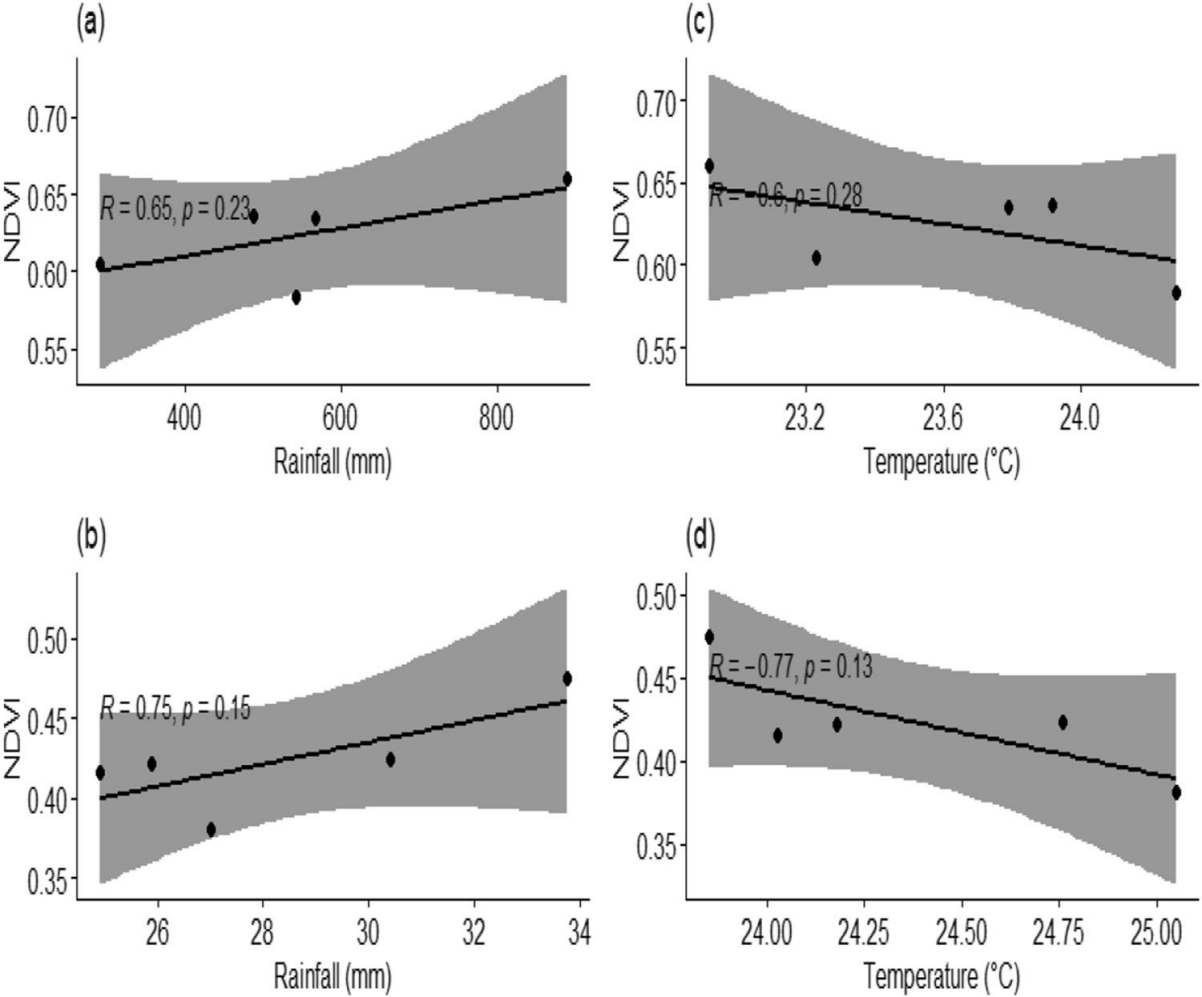
(**a**–**d**) Scatter plot showing correlation between average seasonal sunflower NDVI with temperature and rainfall 2000–2020.

The Pearson correlation was carried out to analyze relationship between climatic variables (rainfall and temperature) versus NDVI per each planting suitability using R studio software. The *r*-coefficient and *p-value* were used to measure the level of statistical relationship and significance. The correlation results are presented in (Fig. 7a–d).

#### Rainfall and NDVI

Findings showed a positive correlation although not statistically significant between sunflower NDVI and rainfall during both seasons January-April and June–September (Fig. 7a,b). The NDVI responded positively to rainfall increase in long and short rainy seasons which indicates positive effects of rainfall with the planting season given the variations. In (Fig. 7a,b), the correlation of NDV and rainfall showed 65% and 75%, respectively implying that the two variables are related to one another during optimal planting seasons. In addition, higher rainfall records was observed during January-April, whereas low rainfall were observed during June–September planting window (see Table 2 and Fig. 7a,b). Variation in rainfall amounts in these two temporal windows was because the higher rainfall occurs during the long term and stable rainy season while the later denotes critical dry season in the year. Using the different modelling approaches in different countries such as Uganda^12^, Sahiwal region in Pakistan^54–57^, in Yamzhog Yumco Basin in South Tibet^70^, Tanzania^49^ and Mongolia China^68^ reported similar findings where high NDVI values was found to correlate with rainy season.

**Table 2:** List of seasonal average rainfall, temperature and NDVI used in this analysis.

January-April				June–September		
Year	Rainfall (mm)	Temp (°C)	NDVI	R (mm)	Temp (°C)	NDVI
2000-1-4	291	23	0.641	27.013	25.05	0.381
2005-1-4	486.788	23.918	0.636	24.905	24.028	0.416
2010-1-4	567.659	23.788	0.635	25.889	24.181	0.422
2015-1-4	542.209	24.277	0.583	30.412	24.76	0.424
2020-1-4	889.444	22.92	0.66	33.763	23.853	0.475

#### Temperature versus NDVI

Negative correlation between NDVI and temperature was observed during January-April and June–September seasons with − 75% and − 77%, respectively (see Fig. 7c and d). Rise in temperature values denote a direct negative response of NDVI in both seasons. This suggests that temperature would not promote suitability in planting season. Unlike rainfall, low temperature values were observed during January-April, whereas higher values were noted during June–September planting season window (see Table 2 and Fig. 7c,d). Variation in records in these two temporal windows was caused by reduced land surface temperature during long term rainy season. On other hand, high temperature records might have been caused by an increase of soil evaporative effects due to higher surface temperature during dry season.

### Conclusion

This paper examined the influence of climatic and environmental variables on suitability of planting season of sunflower in Tanzania. Twelve 4-months moving planting windows were produced for 2000–2001 and 2001–2002. As expected the average suitability of planting season windows in the sunflower growing areas in Tanzania varies depending on climatic parameters especially rainfall seasonal distribution patterns. The planting windows decreases as one moves towards dry season of the year when water shortage becomes critical as result, early planting season windows indicated higher rate of suitability in the study area thus it can be a sign of ensuring higher sunflower crop productivity or yields. Different from rainfall, temperature was found to be optimal over a wider geographical range in Tanzania regardless of the planting season window. Therefore, it is worth noting that temperature rise would mean negative effects to planting season suitability. There was positive and negative relation though not significant between selected climatic variables and NDVI in both high and low or non-suitable planting season windows across twenty years in the study area. Positive response of NDVI to rainfall on long and short rainy seasons indicated good suitability of planting season given the variations. Therefore climatic factors especially rainfall are key determinants in rain-fed agriculture. Future research in this area should explore other specific factors that determine sunflower planting season and growing suitability such as seeds availability, farm size, planting technology, farm management practices and households’ socio-economic characteristics which have not been part of this investigation.

The GEE application platform as applied in this analysis is robust platform that can be used to generate appropriate planting date windows that could help smallholder famers whose reliance on local knowledge and experiences are unreliable and often misleading in this context of climate change and variability. In addition, this could provide farmers with site-specific planting season depending on their variable climates and environments. Hence, we recommend sunflower subsector actors, famers and policy makers to advocate for identification of appropriate season/dates suitable for planting.

## Data Availability

The datasets resulted to these findings of this study are available to the corresponding author of this work upon reasonable request.
